# The Influence of the Type of Electrolyte in the Modifying Solution on the Protective Properties of Vinyltrimethoysilane/Ethanol-Based Coatings Formed on Stainless Steel X20Cr13

**DOI:** 10.3390/ma14206209

**Published:** 2021-10-19

**Authors:** Aleksandra Kucharczyk, Lidia Adamczyk, Krzysztof Miecznikowski

**Affiliations:** 1 Department of Materials Engineering, Faculty of Production Engineering and Materials Technology, Czestochowa University of Technology, Aleja Armii Krajowej 19, 42-200 Czestochowa, Poland; aleksandra.kucharczyk@pcz.pl; 2 Department of Inorganic and Analytical Chemistry, Faculty of Chemistry, University of Warsaw, Ludwika Pasteura 1, 02-093 Warsaw, Poland; kmiecz@chem.uw.pl

**Keywords:** vinyltrimethoxysilane, silane, corrosion, sol–gel

## Abstract

The paper reports the results of the examination of the protective properties of silane coatings based on vinyltrimethoxysilane (VTMS) and ethanol (EtOH), doped with the following electrolytes: acetic acid (AcOH), lithium perchlorate LiClO_4_, sulphuric acid (VI) H_2_SO_4_ and ammonia NH_3_. The coatings were deposited on stainless steel X20Cr13 by the sol–gel dip-coating method. The obtained VTMS/EtOH/Electrolyte coatings were characterized in terms of corrosion resistance, surface morphology and adhesion to the steel substrate. Corrosion tests were conducted in sulphate media acidified up to pH = 2 with and without chloride ions Cl^−^, respectively. The effectiveness of corrosion protection was determined using potentiometric curves. It has been demonstrated that the coatings under study slow down the processes of corrosion of the steel substrate, thus effectively protecting it against corrosion.

## 1. Introduction

The majority of metals in contact with atmospheric air (electrochemical corrosion) form a protective layer—an oxide (passive) film of their surface. The phenomenon of passivation provides a basis for the natural corrosion resistance of some metals and construction alloys, such as aluminium or stainless steels. However, it is not fully sufficient in more aggressive media, for example, in locations, where the metal is exposed to the action of chloride, bromide, or fluorine ions. The effects of corrosion processes are usually associated with additional, often considerable costs; therefore, various methods for protection against corrosion are used [1,2]. As mentioned above, the process of electrochemical corrosion proceeds in the environment of aggressive solutions of electrolytes. Therefore, this phenomenon affects the correct functioning of different types of current sources and materials (such as concrete, steel, etc.), which many people tend to forget.

Stainless steels, or iron alloys containing 12–18% chromium, have for many years enjoyed great popularity because of their corrosion resistance, availability and price. They have unique characteristics, thanks to which they find increasingly wide application in today’s technology. Their corrosion resistance, mechanical or engineering properties enable them to be used in particularly demanding working environments [3]. The martensitic stainless steel X20Cr13 used in the present study, intended for toughening, exhibits high mechanical properties, such as strength, ductility and machinability, while retaining sufficient corrosion resistance. X20Cr13 does not show resistance to chlorine, salts and intercrystalline corrosion. Stainless steel is suitable for operating in the environments of water vapour, low concentrated inorganic acids, solvents and pure water [4]. The corrosion resistance of stainless steels can be enhanced by introducing to them alloy additions, such as nickel or molybdenum, but also by forming protective coats on their surface [5,6].

The modification of metal surface belongs to corrosion prevention methods. Apart from classical metallic, inorganic, or paint coatings, also coatings of organosilicon compounds (siloxane compounds) obtained from modifying solution, attract a lot of attention. These are coatings composed of siloxane bonds formed as a result of the reactions of hydrolysis and condensation. One of the simpler and the most common methods of depositing silane-based coatings is the sol–gel method [7,8]. Silane coatings are used especially for protecting poorly passivating metals, such as Al, Fe, Zn, Mg, Ti and stainless steel [3,9].

Silanes are compounds characterized by low toxicity and, most importantly, offer protection against corrosion and good adhesion to a substrate (e.g., steel). During the deposition of silane-based protective coatings, strong covalent bonds form [5,6]. It has also been shown that organosilanes provide effective corrosion protection of materials, such as aluminium and steel [10,11]. Silanes are used in corrosion protection chiefly as interlayers as they provide the adhesion of coatings to metals [12,13,14,15,16].

The protective properties of silane coatings (structure, stability in time, tightness, corrosion resistance) are related to the parameters of the silane solution (silane type, composition, concentration, pH), as well as with the method of application and the process of drying of the deposited coating at a specific temperature. In many publications [17,18,19,20] the authors, prior to depositing a sol–gel coating, used pre-treatment of the steel surface with acids with the aim of enhancing the adhesion and anticorrosive properties. Coatings based on organosilicon compounds are deposited on the surface of substrate elements, usually from modifying solutions being sol–gel solutions [21,22,23,24,25,26,27,28,29,30,31,32]. In the majority of cases, acetic acid and ammonia were included in the composition of modifying solutions [26,27,28,29,30,31,32].

The precursor of the reaction of synthesis in the sol–gel method are various alcoholates of metals, salts or nitrates. After immersing the metal in a diluted silane solution, particles are adsorbed on the metal surface through hydrogen bonds. The key reactions are hydrolysis and condensation, whereby a compact protective coating forms at the silane/metal interface. The hydrolysis and condensation (polycondensation) reactions occur simultaneously within the whole volume of the solution. The properties of the end product and the rate of the process are strongly influenced by, e.g., the R≡H_2_O: silane mole ratio, medium pH, solvent type, the nature and concentration of catalysts, and temperature; for example, individual stages of the sol–gel process run faster when an appropriate (acidic or basic) catalyst is used [33,34,35].

The present study is devoted to the structural examination and corrosion testing of coatings composed of vinyltrimethoxysilane (VTMS), ethanol and electrolytes, deposited on stainless steel X20Cr13. Within the study, the effect of the addition of an acidic and a basic electrolyte on the structural properties and corrosion protection of the investigated stainless steel was examined. The aim of the investigation was to obtain vinyltrimethoxysilane-based coatings by the dip-coating method of the best possible physical, chemical and anticorrosive protection, which could be used for the corrosion protection of applied metals and their alloys (Fe, Al, Zn, Cu and Cu).

Over a dozen or so years, many papers on the protection of metal surface with silanes have been published; it should be emphasized, however, that those publications did not address the effect of modifiers, i.e., electrolytes of varying pH values, on the process of protecting metals covered with silane coatings against corrosion.

## 2. Materials and Methods

Analytically pure reagents and deionized water were used in experiments. Sol–gel solutions were prepared by mixing vinyltrimethoxysilane (VTMS) of the molecular formula CH_2_=CHSi(OC_2_H_5_)_3_ (supplied by Sigma Aldrich), anhydrous ethyl alcohol EtOH (supplied by Sigma Aldrich) and electrolyte (acidic and basic, respectively). The volumetric VTMS:EtOH:Electrolyte ratio of the obtained coating was 4.84:2.16:3.0.

The following electrolytes were selected for testing:

- acetic acid (AcOH) by Chempur (the method of depositing the coating based on VTMS and acetic acid was developed in previous studies [36]);
- lithium perchlorate (LiClO_4_) by Fluka Chemika, as a strongly oxidizing anion of inhibiting properties [37,38,39,40,41,42];
- sulphuric acid (VI) (H_2_SO_4_) by Chempur; an acid medium, with a passive film forming on the metal surface, containing sparingly soluble thermodynamically stable oxidation products [43,44,45,46];
- ammonia (NH_3_)- P.P.H. by Polskie Odczynniki Chemiczne; a basic medium, for controlling the hydrolysis and condensation rates [47,48,49,50,51,52].

### 2.1. The Influence of the Reaction Environment on the Sol–Gel Process

Four (4) electrolytes (acetic acid CH_3_COOH (AcOH), lithium perchlorate LiClO_4_, sulphuric acid (VI) H_2_SO_4_ and ammonia NH_3_) were chosen for the sol–gel process on account of the hydrolysis reaction: acidic and basic hydrolysis.

Table 1 shows the effect of the medium (electrolyte) on individual stages of the sol–gel process. Table 2 gives examples of substances that speed up this process.

The sol–gel process can be run by two methods. The first method is one-stage basic or acidic catalysis. In the case of basic catalysis, hydrolysis proceeds with the participation of the hydroxyl anion (OH^−^). This ion reacts directly with the silicon atom, leading to the formation of silanol and the RO^−^ group. It enables semi-transparent gels of high porosity to be obtained. In the case of acidic catalysis, on the other hand, the hydrolysis process is initiated by acid. Protons H^+^ react with the oxygen atoms bonded with the silicon atom in the –OR or OH group. This causes the electron cloud to shift towards the oxygen atom in the Si-O bond. As a consequence, this results in an increase in positive charge on the silicon atom. The water molecule combines with the silicon atom, which is followed by disintegration and the formation of silanols and alcohol. In acidic catalysis, we obtain transparent gels of low porosity [50].

For technological reasons, the sol–gel process is most favourably carried out by using the second of the above-mentioned methods, i.e., two-stage acidic-basic catalysis. This shortens the time necessary for obtaining the gel. The method comprises the first stage—hydrolysis at pH < 7, the second stage—raising the medium reaction up to a pH of approximately 7, whereby we slow down the hydrolysis process and accelerate the gelation process [51].

### 2.2. Preparation of Test Material

Stainless steel X20Cr13 of the following composition (in wt%): C-0.17; Cr-12.6; Si-0.34; Ni-0.25; Mn-0.30; V-0.04; P-0.024; and S < 0.005 was used for testing. Test samples were in the shape of 5 mm-diameter cylinders. Their walls were isolated in polymethyl methacrylate frames using epoxy resin. The geometric working surface area of the samples was 0.196 cm^2^. Prior to experiments, the samples were each time mechanically polished on abrasive papers with a decreasing grit size up to grade 2000, and then rinsed with distilled water and ethyl alcohol. Before applying a coating, each sample was flushed with acetone to degrease its surface. On the prepared electrodes, coatings were deposited by the dip-coating method following the procedure developed in the paper [36]. The composition and the stirring parameters for the test coatings are shown in Table 3.

The anticorrosive properties of the obtained coatings were assessed in two corrosion media: 0.5 mol dm^−3^ Na_2_SO_4_ (pH = 2) and 0.5 mol dm^−3^ Na_2_SO_4_ + 0.5 mol dm^−3^ NaCl (pH = 2) using a potentiodynamic technique with the use of a scanning potential from—0.8 V to 1.6 V at a polarization rate of 10 mVs^−1^. The values of all potentials were measured and expressed relative to the saturated calomel electrode.

The composition and surface appearance of the coatings deposited on the investigated steel were assessed using a JEOL JSM-6610 LV scanning electron microscope with an EDS-type X-ray microanalyzer (JEOL, Tokyo, Japan). Microstructural examination was carried out with a KEYENCE VHX 7000 digital microscope and an Olympus GX41 optical microscope (Keyence, Mechelen, Belgium). The surface roughness of the coatings was measured using an Hommel Tester T1000 profilometer (JENOPTIK Industrial Metrology, Jena, Germany). The microscopic surface maps were made using an AFM (Atomic Force Microscope) NanoScope V MultiMode 8 (BRUKER, Bremen, Germany). The characteristics of the coatings were determined using Attenuated Total Reflection Fourier Transform Infrared Spectroscopy (ATR- FTIR) Bruker Optics-Vertex 70 V (BRUKER, Bremen, Germany). The coatings thicknesses were measured using a DT-20 AN 120 157 meter (ANTICORR, Gdańsk, Poland). Electrochemical measurements were taken in the classical three-electrode system using a CHI 706 measuring station (CH Instruments, Austin, TX, USA). The working electrodes were steel X20Cr13 coated and uncoated as well as glassy carbon, an auxiliary electrode (platinum), and a reference electrode (the saturated calomel electrode, SCE).

The adhesion of the test coatings was assessed by a qualitative test using Scotch^TM^ Tape (Scotch^TM^ Brand, St. Paul, MI, USA).

### 2.3. The Preparation of Samples for Structural Examination (Sample Cross-Section)

Samples for structural examination (sample cross-section) were prepared in four steps: application of a coating on steel, cutting the sample through in the plane perpendicular to the surface, then the samples were embedded in epoxy resin with graphite. So embedded specimens were subjected to grounding and polishing to obtain a mirror surface.

### 2.4. Corrosion Resistance Test in a Potassium Hexacyanoferrate (III) Solution (Ferroxyl Test)

For performing a quick test to indicate the barrier nature of the proposed protective coatings, it was applied the well-known and widely used ferroxyl test (ferroxyl indicator) in a modified form. For this purpose, the tested plate was immersed in a solution containing potassium ferricyanide. In the presence of iron ions (formed in the process of corrosion in an acid medium), insoluble blue iron (II) hexacyanoferrate (III) (Prussian Blue) appears, indicating that the material dissolving in anodic locations. A 2-mmol dm^−3^ potassium hexacyanoferrate (III) K_3_[Fe(CN)_6_] solution was utilized for testing. An X20Cr13 steel sample uncovered and covered with a VTMS/EtOH/AcOH coating in a VTMS concentration of 3.16 mol dm^−3^ were immersed in potassium hexacyanoferrate (III) solution. Afterward, electrochemical measurements were taken by the cyclic voltammetry in the potential range from −0.6 V to 1.2 V vs. SCE [49,50].

## 3. Results

### 3.1. Microstructural Observations and Chemical Analysis

**Figure 1:**
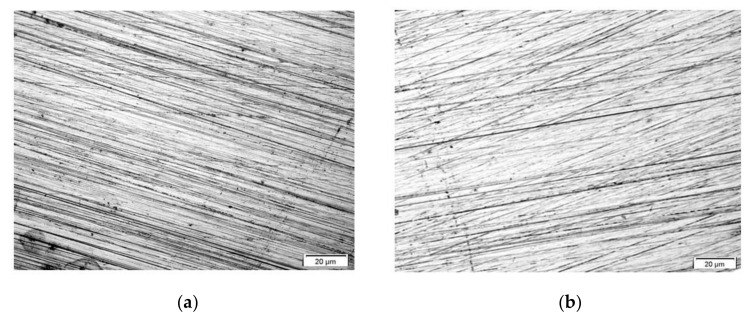

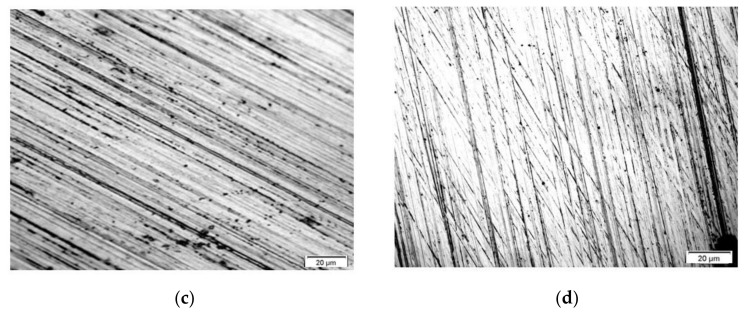
Coatings topography: VTMS/EtOH/AcOH (**a**), VTMS/EtOH/LiClO_4_ (**b**), VTMS/EtOH/H_2_SO_4_ (**c**), VTMS/EtOH/NH_3_ (**d**), (×200).

**Figure 2:**
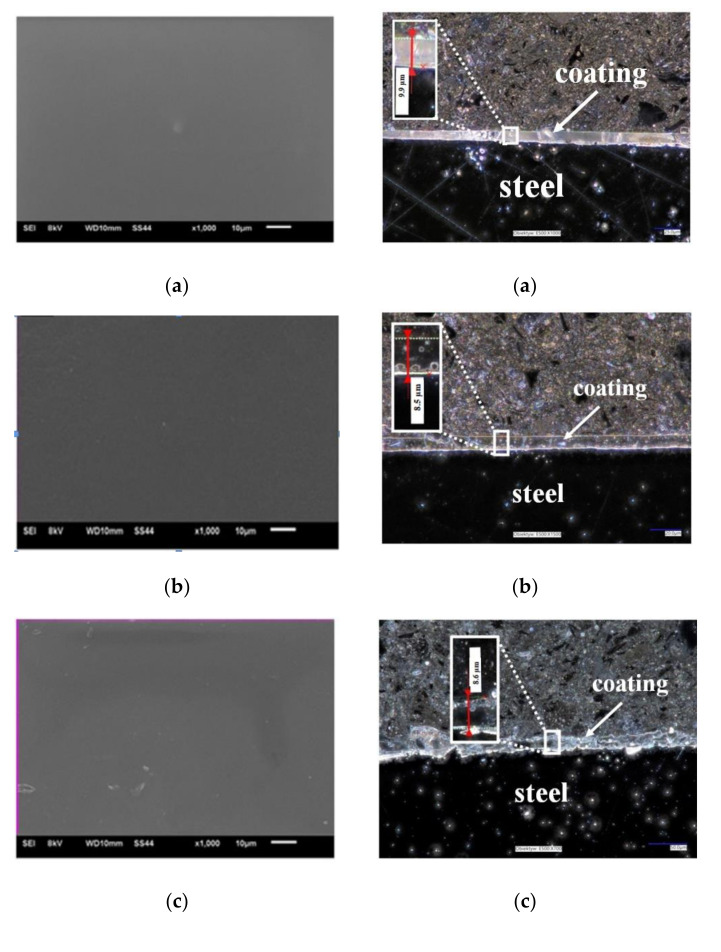

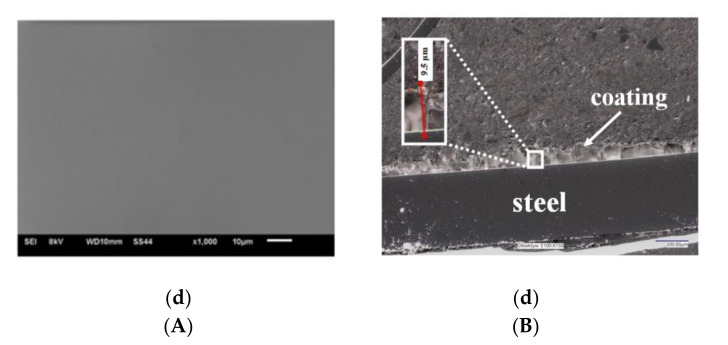
(**A**) Topography of VTMS coatings deposited on steel X20Cr13 (SEM Jeol JSM-6610 LV), (**B**) Cross-section of coatings deposited on steel X20Cr13 (digital microscope KEYANCE VHX 7000, ×200). Coatings: VTMS/EtOH/AcOH (**a**), VTMS/EtOH/LiClO_4_ (**b**), VTMS/EtOH/H_2_SO_4_ (**c**), VTMS/EtOH/NH_3_ (**d**).

The topography of obtained coatings was assessed using a light microscope. Figure 1 and Figure 2 show the morphology of the investigated coatings deposited on the X20Cr13 stainless steel surface. All of the four coating types uniformly cover the entire surface of the electrodes without any free structural spaces. A structure of the metallic substrate with polishing traces is visible in the photographs (Figure 1). As shown in Figure 1, the coatings morphology is compact, smooth, shiny and transparent over the entire sample surface.

Figure 2 represents the topography of VTMS/EtOH/AcOH, VTMS/EtOH/LiClO_4_, VTMS/EtOH/H_2_SO_4_, VTMS/EtOH/NH_3_ coatings, revealed using a scanning electron microscope. The obtained results confirm the previous observations: regardless of the added electrolyte, the morphology of VTMS coatings covers uniformly the sample surface, forming a compact, tight and homogeneous structure.

Figure 2B shows the cross-section of the obtained VTMS/EtOH/Electrolyte coatings deposited on the X20Cr13 stainless steel. The recorded profile indicates that the coatings are uniformly deposited on the steel surface. The structural observations confirm that the coatings are free from any cracking and their surface roughness is negligible, which is a huge asset from the point of view of the corrosion resistance of steel. The average coatings thickness (as measured in four locations on the sample) were the following for respective coatings: VTMS/EtOH/AcOH 11.4 µm (a); VTMS/EtOH/LiClO_4_ 8.05 µm (b); VTMS/EtOH/H_2_SO_4_ 8.65 µm (c); VTMS/EtOH/NH_3_ 12.8 µm (d). Based on the cross-section, it was demonstrated that the modification of the silane solution with various electrolytes had a significant effect on the coating thickness.

#### 3.1.1. Chemical Composition of the VTMS/EtOH/Electrolyte Coatings

The chemical composition of the produced coatings was determined using an electron scanning microscope equipped with an EDS X-ray chemical analyzer. Based on the performed chemical analysis, the contents of silicon for respective coatings are as follows: VTMS/EtOH/AcOH 32.47%; VTMS/EtOH/LiClO_4_ 29.05%; VTMS/EtOH/H_2_SO_4_ 30.26%; VTMS/EtOH/NH_3_ 25.87%. The rest was made up of the elements C and O.

#### 3.1.2. Testing for Adhesion to the Substrate

Immediately after depositing VTMS/EtOH/Electrolyte coatings, their adhesion to the X20Cr13 stainless steel substrate was tested using Scotch^TM^ Tape. The produced coatings are characterized by good adhesion to the steel substrate.

#### 3.1.3. Surface Roughness of Obtained Coatings

**Figure 3:**
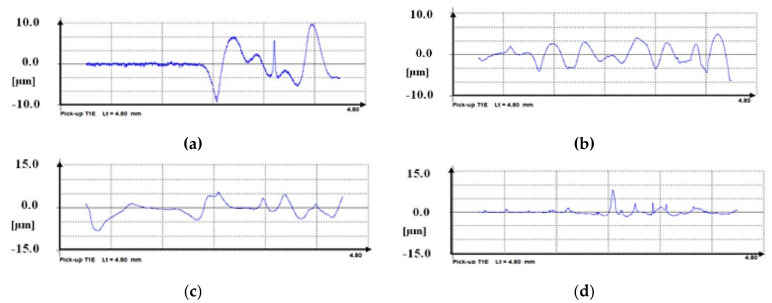
Roughness measurement Ra for coatings deposited on X20Cr13 steel: VTMS/EtOH/AcOH (**a**), VTMS/EtOH/LiClO_4_ (**b**), VTMS/EtOH/H_2_SO_4_ (**c**), VTMS/EtOH/NH_3_ (**d**). Profilometer Hommel Tester T1000.

The measurement of the surface roughness of the VTMS/EtOH/Electrolyte coatings deposited on the X20Cr13 stainless steel taken with a profilometer is represented in Figure 3. Both the topography and profile of the coatings confirm that the coatings are free from cracking, and their surface roughness is little (low Ra values). The testing results differ, depending on the electrolyte used; the closest Ra values can be observed for VTMS/EtOH/AcOH and VTMS/EtOH/NH_3_ coatings (Ra = 0.40–0.43 µm). Table 4 shows the results of the measurement of parameter Ra.

**Figure 4:**
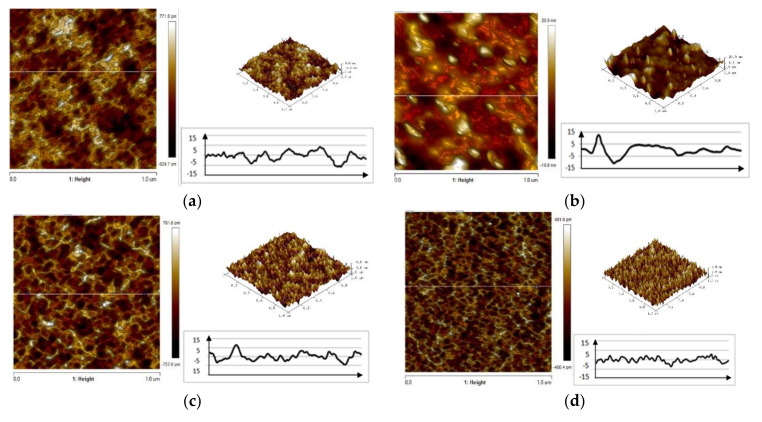
AFM images of the surface of coatings deposited od steel X20Cr13: VTMS/EtOH/AcOH (**a**), VTMS/EtOH/LiClO_4_ (**b**), VTMS/EtOH/H_2_SO_4_ (**c**), VTMS/EtOH/NH_3_ (**d**). Pictures were taken using an AFM NanoScope V MultiMode 8 Bruker.

The examination made using an AFM microscope confirms the previous findings that the addition of electrolyte has an effect on the coating surface roughness. The surface morphologies of VTMS/EtOH/Electrolyte coatings produced on the metal surface, with a varying electrolyte addition, are illustrated in Figure 4. The recorded values of parameter Ra for respective coatings are as follows: VTMS/EtOH/AcOH 0.381 µm; VTMS/EtOH/LiClO_4_ 0.908 µm; VTMS/EtOH/H_2_SO_4_ 1.45 µm; VTMS/EtOH/NH_3_ 0.389 µm.

Protective coatings are generally porous layers; after some time, the surface of steel or metal will come into contact with an aggressive electrolyte solution, water, or oxygen molecules. A discontinuity in the coating may initiate pitting or crevice corrosion. Obtained protective coatings, as compared to the protected elements, are usually extremely thin.

#### 3.1.4. Thickness of Obtained Coatings

One of the key parameters influencing the corrosion resistance of elements is the thickness of their protective coatings. In the present study, this parameter has been analyzed using three examination methods. Based on profile examination (Figure 2B), the thickness of obtained coatings was analyzed. The thickness of each coating is the average of 4 measurements: VTMS/EtOH/AcOH 11.4 µm (a); VTMS/EtOH/LiClO_4_ 8.05 µm (b); VTMS/EtOH/H_2_SO_4_ 8.65 µm (c); VTMS/EtOH/NH_3_ 12.8 µm (d).

The recorded thicknesses measured with a profilometer are given in Table 5.

To compare the thicknesses of the coatings, in addition to the methods described above, thickness measurements were taken using a DT-20 Testan meter with an integrated probe designed for measuring on ferro- and non-ferromagnetic substrates. A series of 10 measurements (at different locations on the sample) was done; Table 6 provides recorded thickness values for VTMS/EtOH/Electrolyte coatings. The obtained coatings thickness values are consistent with those produced with a digital microscope and a profilometer.

Based on the performed measurements using three instruments (a digital microscope, profilometer, and a thickness meter), the mean coatings thickness was determined (Table 7).

The differences in coatings thickness between individual methods were negligible, which confirms the usefulness of the applied methods for coatings thickness measurement.

### 3.2. Analysis of Coatings Composition

**Figure 5:**
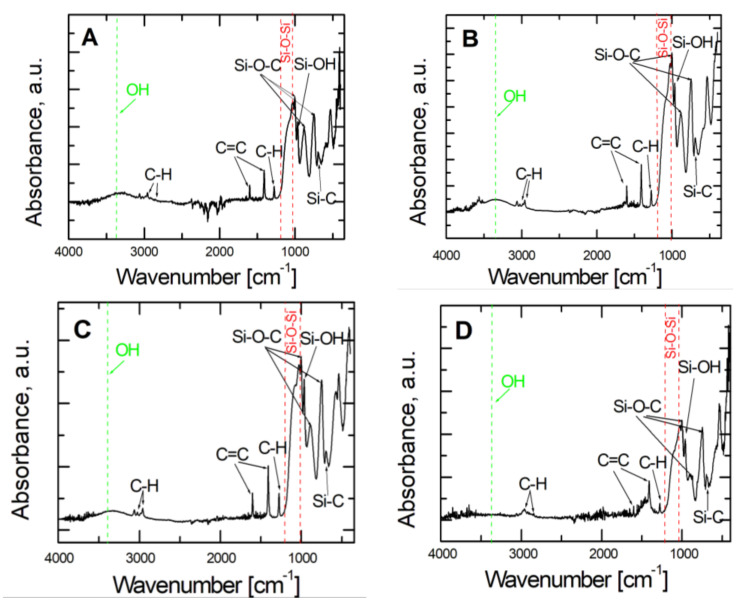
ATR- FTIR spectra obtained for VTMS coatings with a concentration of 3.16 mol dm^−3^ deposited on steel X20Cr13: VTMS/EtOH/AcOH (**A**), VTMS/EtOH/LiClO_4_ (**B**), VTMS/EtOH/H_2_SO_4_ (**C**), VTMS/EtOH/NH_3_ (**D**).

The characteristics of vinyltrimethoxysilane- based coatings deposited on the X20Cr13 steel substrate were determined using Attenuated Total Reflection Fourier Transform Infrared Spectroscopy (ATR-FTIR). The ATR-FTIR spectra of VTMS/EtOH/Electrolyte coatings are shown respectively in Figure 5. Characteristic absorption peaks were observed for VTMS in the range of 4000–400 cm^−1^. The absorption bands observed at the values of 2953 cm^−1^, 2857 cm^−1^ and 1290 cm^−1^ correspond to the asymmetric tensile and bending vibrations of the C-H bond belonging to the –Si-(OCH_3_) group. Subsequent peaks were noted at the values of 1602 cm^−1^ and 1410 cm^−1^, which correspond to the tensile vibrations of the C=C bond of the CH_2_=CH- group. The bands of values of 1000 cm^−1^, 883 cm^−1^ and 750 cm^−1^ correspond to the vibrations of Si-O-C. The wide band occurring at about 1100 cm^−1^ corresponds to the asymmetric tensile vibrations of the Si-O-Si bond. The peak at the value of 704 cm^−1^ corresponds to the Si-C bond. The wide absorption band at about 3400 cm^−1^ was caused by the –OH groups. The peak at 964 cm^−1^ was ascribed to the asymmetric bending vibrations of the Si-OH bond, whereas the band observed at the wavelength of 3053 cm^−1^ matches the vibrations in the alkene group.

### 3.3. Electrochemical Analysis

To make the assessment of the kinetic tendency to either general or pitting corrosion, measurements of open circuit potential (OCP) were taken for steel uncovered and covered with a VTMS coating, respectively, with the addition of electrolyte in the form of either: acetic acid (b), lithium perchlorate (c) sulphuric acid (VI) (d), or ammonia (e).

**Figure 6:**
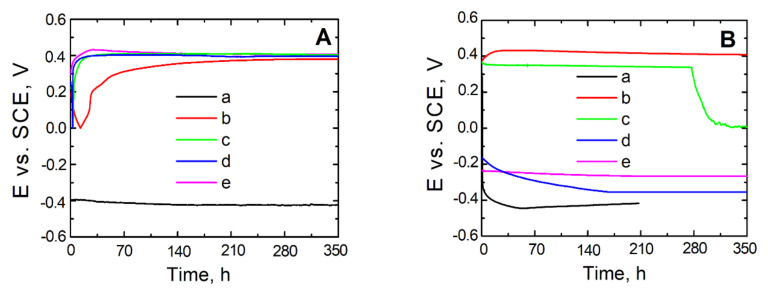
Potential measurement in open circuit potential OCP from exposure time in solution: 0.5 mol dm^−3^ Na_2_SO_4_ mol dm^−3^ pH = 2 (**A**) and 0.5 mol dm^−3^ Na_2_SO_4_ + 0.5 mol dm^−3^ NaCl pH = 2 (**B**) for steel X20Cr13 uncovered (a) and covered with coatings VTMS/EtOH: CH_3_COOH (b), LiClO_4_ (c), H_2_SO_4_ (d), NH_3_ (e).

As shown by the open circuit potential measurements illustrated in Figure 6A,B, the steel not covered with a coating directly after being immersed in the test solutions exhibits a potential of approximately −0.4 V, which decreases for longer exposure times and takes on the value of corrosion potential for steel (−0.5 V). For steel X20Cr13 covered with the coatings: VTMS/EtOH/LiClO_4_, VTMS/EtOH/H_2_SO_4_ and VTMS/EtOH/NH_3_, Figure 6A, the OCP potential increases for the initial 24 h until reaching a value of 0.4 V. As can be seen from Figure 6A (line b), the steel covered with a VTMS/EtOH/AcOH coating directly after being immersed in the corrosion solution shows a potential of 0.2 V, which increases up to 0.4 V after 24 h exposure. The values of potential for all steels covered with coatings after prolonged immersion in the corrosion solution show potential from the passive range, so more positive than E*_kor_* (0.5 V).

The dependence of the open circuit potential of uncoated and coated steel on the time of holding in the chloride ion-containing corrosion solution is represented in Figure 6B. The uncoated X20Cr13 steel undergoes active dissolution after approximately 50 h of immersion in the corrosion solution. By contrast, the steel covered with VTMS-based coatings, upon immersion in the corrosion solution, exhibits a potential from the passive range. The potential of the steel covered with VTMS/EtOH/AcOH coatings increases, for the initial 24 h, up to a value of approximately 0.45 V and stays on this level for another 13.5 days; for VTMS/EtOH/H_2_SO_4_, the potential is −0.25 V and remains for 350 h; for VTMS/EtOH/NH_3_, after 150 h, it amounts to −0.35 V and holds on this level for subsequent 200 h; and for VTMS/EtOH/LiClO_4_, the potential stays at the level of 0.35 V for 240 h and then dramatically decreases to a value of 0.0 V.

It is worth noting that the stationary potential value of the coated steel, despite the log time of exposure in the chloride ion-containing corrosion solution, is more positive than the stationary potential value of steel. Microscopic observations after the measurement did not reveal any local corrosion effects under the VTMS/EtOH/AcOH coating, which indicates significant substrate protection.

**Figure 7:**
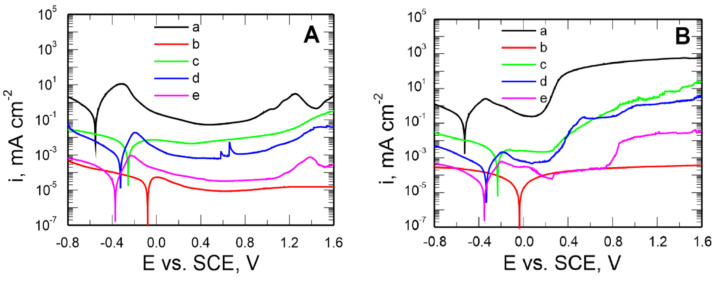
Potentiodynamic polarization curves recorded in the solution: 0.5 mol dm^−3^ Na_2_SO_4_ pH = 2 (**A**) and 0.5 mol dm^−3^ Na_2_SO_4_ + 0.5 mol dm^−3^ NaCl pH = 2 (**B**) for uncoated steel X20Cr13 (a) and covered with coatings VTMS concentrations in a 3.16 mol dm^−3^ solution and the addition of an electrolyte: CH_3_COOH (b), LiClO_4_ (c), H_2_SO_4_ (d), NH_3_ (e). Polarization rate 10 mVs^−1^, solutions in contact with air.

To establish the most effective influence of electrolytes on the anticorrosion properties of the produced VTMS silane coatings deposited on the X20Cr13 steel, the assessment of their capacity for inhibiting general and pitting corrosion was made using potentiodynamic curves. The experiment was conducted in two solutions:

- for general corrosion: 0.5 mol dm^−3^ Na_2_SO_4_ pH = 2 (Figure 7A),
- for pitting corrosion: 0.5 mol dm^−3^ Na_2_SO_4_ + 0.5 mol dm^−3^ NaCl pH = 2 (Figure 7B).

The potential range of −0.8–1.6 V for the X20Cr13 steel uncoated and coated, respectively.

As follows from Figure 7A, the produced VTMS/EtOH/Electrolyte coatings inhibit the cathodic and anodic processes and shift the corrosion potential of the steel by approximately 0.5 V (the VTMS/EtOH/AcOH coating). The anodic current densities for the steel covered with VTMS/EtOH/Electrolyte coatings in the passive range are smaller by 1–4 times than those for the uncoated steel.

To assess the capacity of the produced coatings to inhibit pitting corrosion, similar potentiodynamic curves were plotted for a sulphate solution acidified to pH = 2, containing an addition of 0.5 mol dm^−3^ of chloride ions (Figure 7B).

The corrosion potential of the X20Cr13 steel for all coatings is shifted by approximately 0.1–0.5 V towards positive values relative to the corrosion potential values recorded for the uncoated steel (E*_kor_* = −0.527 V). Lower values of cathodic and anodic current densities were also observed for the steel covered with these coatings, compared to the uncoated steel.

The shape of the polarization curves shows that the pitting nucleation potential (E_pit_) amounts to, respectively: for the uncoated steel 0.12 V; for the steel covered with the coatings: VTMS/EtOH/LiClO_4_ 0.18 V; VTMS/EtOH/H_2_SO_4_ 0.19 V; VTMS/EtOH/NH_3_ 0.64 V. The thermodynamic susceptibility to pitting is similar for the coatings VTMS/EtOH/LiClO_4_ and VTMS/EtOH/H_2_SO_4_. As shown by Figure 7B (line b), in the case of employing the VTMS/EtOH/AcOH coating for steel protection, no puncture potential of the passive film (pitting nucleation potential) was observed. The silane coating modified with acetic acid effectively hinders the access of aggressive anions to the steel substrate, thus protecting the substrate against pitting corrosion. Microscopic observations after the measurement did not reveal any local corrosion effects under the VTMS/EtOH/AcOH coating. Figure 7B implies that the application of coatings on steel protects the substrate against local corrosion.

To verify the resistance of coatings deposited on steel to pitting corrosion, the chronoamperometric method was employed. In this method, variations in current density are recorded as a function of time after applying a constant potential to the working electrode. From chronoamperometric curves, one can infer the nucleation of pits.

To determine the stability of the applied coats, the time of holding the test samples in the corrosion solution containing chloride ions and the value of current density were compared at a preset potential. Chronoamperometric curves were recorded in a 0.5 mol dm^−3^ solution of Na_2_SO_4_ + 0.5 mol dm^−3^ NaCl with pH = 2 at a potential of 0.1 V for uncoated and coated steel, respectively.

**Figure 8:**
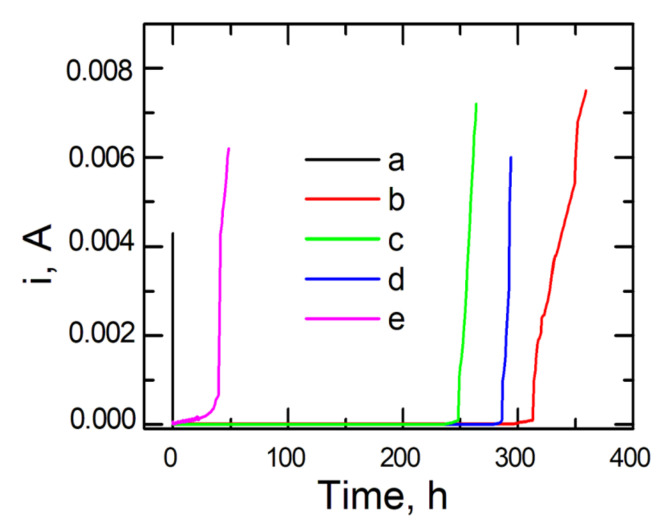
Chronoamperometric curves recorded in a chloride solution (0.5 mol dm^−3^ Na_2_SO_4_ + 0.5 mol dm^−3^ NaCl pH = 2) for X20Cr13 steel not covered with the coating (a) and coated with VTMS in 3.16 mol dm^−3^ solution and addition of electrolyte: CH_3_COOH (b), LiClO_4_ (c), H_2_SO_4_ (d), NH_3_ (e).

Figure 8 shows the chronoamperometric curves for steel plotted at a potential of 0.1 V red out from the polarization curves, Figure 7B. As can be observed, the initiation of pit formation on the steel occurs within several seconds, after which the value of current density dramatically increases. In the case of applying the following coating types, VTMS/EtOH/AcOH, VTMS/EtOH/LiClO_4_, and VTMS/EtOH/H_2_SO_4_, the highest corrosion resistance was achieved. The increase in current density for the above-mentioned coatings occurred within a time span ranging from 250 to 312 h. The best capacity to block the transport of chloride ions responsible for pitting corrosion is shown by the VTMS/EtOH/AcOH coating (312 h).

### 3.4. Corrosion Resistance Test in a Potassium Hexacyanoferrate (III) Solution (Ferroxyl Test)

To demonstrate the corrosion resistance of coatings deposited on the X20Cr13 steel, electrochemical tests were carried out in a 2 mmol dm^−3^ solution of K_3_[Fe(CN)_6_].

**Figure 9:**
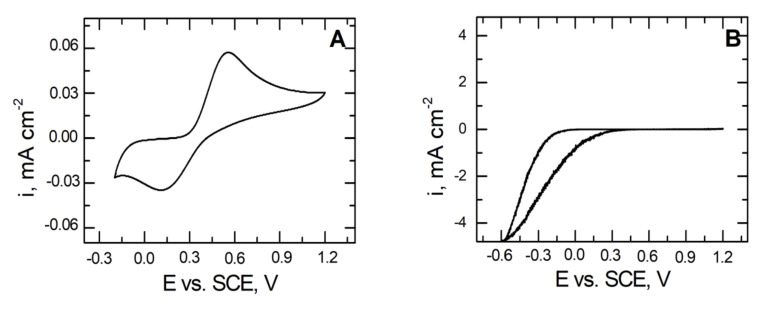
Voltammetric response for: glassy carbon (**A**) and coated X20Cr13 steel with VTMS/EtOH/AcOH (**B**). Electrolyte: 2 mmol dm^−3^ K_3_[Fe(CN)_6_]. Polarization rate 10 mVs^−1^.

Figure 9 shows a typical voltammetric response of the glassy carbon electrode (A) and the VTMS/EtOH/AcOH–coated X20Cr13 steel electrode (B) in the presence of Fe(CN)_6_^3−^ sampler ions. In the case of the pure glassy carbon electrode (Figure 9A), we observe a well-developed and quasi-reversible pair of ferrocyanide ions. By contrast, Figure 9B illustrates the voltammetric response of the VTMS/EtOH/AcOH coating on the X20Cr13 steel substrate, on which it does not observe any electrochemical response in the investigated potential range. This is associated primarily with the fact that ferrocyanide ions do not cross through the produced VTMS/EtOH/AcOH layer (pores in the layer are smaller than the size of the ferrocyanide ion). Additionally, no formation of the blue colouring (Prussian Blue formation) was observed on the steel surface, confirming definitely that the obtained coating provides a compact and tight protective barrier. Moreover, the VTMS/EtOH/AcOH layer formed on the X20Cr13 steel blocked the transport of electrons to ferrocyanide ions, has manifested itself by the attenuation of the redox currents (Figure 9B) [49,50].

## 4. Conclusions

The investigation of VTMS/EtOH/Electrolyte coatings has shown that the sol–gel process can be used for producing protective layers on stainless steel X20Cr13.

The selection of the appropriate electrolyte has a significant impact on the corrosion and structural properties of VTMS coatings (a uniform surface with no visible defects in the structure). The produced coatings exhibit good adhesion to the substrate and, in addition, extend the duration of steel resistance to the action of chloride and sulphate ions in an acid medium. The best ability to block the transport of chloride ions responsible for the pitting corrosion of steel is shown by the VTMS/EtOH/AcOH coating. The surface roughness and thickness of the coating may be influenced by the size of the doped electrolyte ion. Acetic acid-doped silane coatings deposited on the X20Cr13 steel, with low surface roughness and a small thickness of the coating, exhibit the anticorrosion properties.

Data obtained from potentiodynamic measurements show that the produced VTMS/EtOH/Electrolyte coatings provide stainless steel's anodic and barrier protection. An experiment using a potassium hexacyanoferrate (III) solution has confirmed that the VTMS/EtOH/AcOH coating forms a uniform, tight structure and blocks the transfer of electrons to ferrocyanide ions.

## Figures and Tables

**Table 1:** The influence of the reaction environment on the speed of the sol–gel process.

Reaction Environment	Hydrolysis	Condensation	Gelation
alkaline	speed drop	high speed	fast
inert	the slowest	faster	easier at pH ≥ 7
acidic	high speed	0–2 pH—high speed 2–3 pH—the slowest 3–5 pH—speed drop	slow

**Table 2:** Substances accelerating the sol–gel process.

Stages of the Sol–Gel Process	Hydrolysis	Condensation
Catalysts	Aqueous solution HCl, HNO_3_, HF, icy CH_3_COOH	NH_3_, NaOH

**Table 3:** Composition of coatings and mixing parameters.

Number	C_m_ VTMS [mol dm^−3^]	EtOH [mL]	H_2_O [mL]	Electrolyte	Electrolyte Concentration [mol dm^−3^]	Mixing Time [h]	Immersion Time of the Steel Sample [min]	Rotations/min
1	3.16	2.16	2.9833	CH_3_COOH 0.0167 mL	0.003	48	20	400–1000
2	3.16	2.16	2.8936	LiClO_4_ 0.1064 g	0.1	48	20	400–1000
3	3.16	2.16	2.5	2 mol dm^−3^ H_2_SO_4_ 0.5 mL	0.1	24	20	400–1000
4	3.16	2.16	1–2 drops of 25% ammonia were added to 100 mL of water, then the pH was measured with a litmus paper		pH = 8–9	24	20	400–1000

**Table 4:** Roughness parameter Ra for individual coatings deposited on steel X20Cr13.

Coating	Ra [µm]
VTMS/EtOH/AcOH	0.40
VTMS/EtOH/LiClO_4_	0.87
VTMS/EtOH/H_2_SO_4_	1.32
VTMS/EtOH/NH_3_	0.43

**Table 5:** Thickness measurement results for individual coatings on steel X20Cr13.

Coating	Coating Thickness [µm]
VTMS/EtOH/AcOH	10.3
VTMS/EtOH/LiClO_4_	7.9
VTMS/EtOH/H_2_SO_4_	8.8
VTMS/EtOH/NH_3_	11.4

**Table 6:** Coating thickness measurement results using a gauge Testan DT-20.

	Coatings Thickness [µm]			
	VTMS/EtOH/ AcOH	VTMS/EtOH/ LiClO_4_	VTMS/EtOH/ H_2_SO_4_	VTMS/EtOH/ NH_3_
**Average**	9.6	8.5	8.4	11.5

**Table 7:** The average thickness of the coatings calculated by measurements from three instruments (a digital microscope, profilometer, and a thickness meter).

Coating	Digital Microscope Average [µm]	Profilometer Average [µm]	Thickness Meter Average [µm]	Thickness of Coatings (Averegae of the Three Devices) [µm]
**VTMS/EtOH/AcOH**	11.4	10.3	9.6	**10.4**
**VTMS/EtOH/LiClO_4_**	8.05	7.9	8.5	**8.2**
**VTMS/EtOH/H_2_SO_4_**	8.65	8.8	8.4	**8.6**
**VTMS/EtOH/NH_3_**	12.8	11.4	11.5	**11.9**

## Data Availability

Not applicable.
